# Extracellular Traps Increase Burden of Bleeding by Damaging Endothelial Cell in Acute Promyelocytic Leukaemia

**DOI:** 10.3389/fimmu.2022.841445

**Published:** 2022-04-11

**Authors:** Yufeng Wang, Chunxu Wang, Nan Zuo, Hao Yang, Shaohong Fang, Jialan Shi

**Affiliations:** ^1^ Department of Hematology, First Affiliated Hospital of Harbin Medical University, Harbin, China; ^2^ The Key Laboratory of Myocardial Ischemia, Ministry of Education, Harbin, China; ^3^ Department of General Surgery, Second Affiliated Hospital of Harbin Medical University, Harbin, China; ^4^ Department of Cardiology, Second Affiliated Hospital of Harbin Medical University, Harbin, China; ^5^ Departments of Research and Medical Oncology, Veterans Affairs (VA) Boston Healthcare System, Dana-Farber Cancer Institute, and Harvard Medical School, Boston, MA, United States

**Keywords:** extracellular traps, endothelial cell, acute promyelocytic leukaemia, bleeding, platelets

## Abstract

The rate of complete remission of acute promyelocytic leukemia (APL) is currently over 90% because of the use of all-trans retinoic acid (ATRA) with arsenic trioxide (ATO). However, hemorrhagic mortality has emerged as the most significant barrier to APL-induced remission. Neutrophils extracellular traps (NETs/ETs) cause vascular leakage by damaging the integrity of endothelial cells. We have previously demonstrated that APL cells treated with ATRA/ATO undergo a cell death process, releasing extracellular chromatin, termed ETosis/NETosis. However, the mechanism underlying the involvement of ETs in endothelial injury in APL remain largely unknown. Here, we analysed the ability of mature and immature neutrophils to release ETs, and their interaction with platelets (PLTs) in APL. Importantly, the effect of ETs on vascular endothelium in APL was discussed. Our results showed that the ability of immature neutrophils to release ETs was impaired in APL, whereas mature neutrophils produced ETs, which were associated with activated PLTs. Moreover, ATRA+ATO induced immature neutrophil differentiation, as well as increased the release of ETs from mature neutrophils. The excessive ETs damaged endothelial cells, causing blood cell leakage. Removing ETs using DNase 1 alleviated endothelial damage and improved blood cells leakage. Our results indicate that vascular endothelial injury is at least partially associated with ETs in APL, and that targeting ETs production may be an effective approach for relieving vascular leakage and reducing the burden of bleeding in APL.

## Introduction

Haemorrhage is the most prevalent cause of induction deaths of APL, accounting for more than half of all induction deaths (1). Heparin, antifibrinolytic drugs, and platelets (PLTs) transfusion failed to significantly reduce this high death burden (2). Therefore, it is imperative to further understand the pathogenesis of APL early hemorrhage. Early bleeding has been found to be markedly correlated with an increase in white blood cell count (3). Activated neutrophils release intracellular chromatin and nuclear contents to the outside of the cell in response to various stimuli, forming a network called neutrophil extracellular traps (NETs/ETs) (4). ETs consists of decondensed chromatin structure, decorated with antimicrobial proteins and enzymes such as histone and neutrophil elastase, which play a role in capturing and destroying pathogens (5). Although ETs plays an important role in host defense, its harmful effects have been gradually discovered. ETs can damage organs through direct cell damage and uncontrolled inflammatory response in diseases such as sepsis, acute lung injury and acute liver injury (5). The active components of ETs promote coagulation, interfere with the anticoagulation system, and provide a scaffold for PLTs and erythrocytes adhesion and fibrin deposition to facilitate thrombosis (6). Studies have found that coronary thrombi contain a large amount of ETs, and the increase of ETs level is related to the infarct size of acute myocardial (7). ETs is also involved in the pathogenesis of cancer. ETs change the adhesion of cancer cells, promote epithelial-mesenchymal transformation and enhance inflammation in the microenvironment to potentiate metastasis (8). ETs can also awaken dormant cancer cells, leading to tumor recurrence and dissemination (9). Moreover, the occurrence and development of metabolic diseases and autoimmune diseases are also related to the increase of ETs (4). However, relatively little is known about the formation of ETs in leukemia, especially APL.

Compared with solid tumors, the role of ETs in leukemia is still not fully understood. With the in-depth study of ETs, the enhanced ability of neutrophils to form ETs has been found in patients with chronic lymphoblastic leukemia (CLL) and chronic myeloid leukemia (CML). Neutrophils isolated from newly diagnosed CML patients showed the increased of ETs formation after treated with ionomycin and phorbol 12-myristate 13-acetate (PMA) (10). Neutrophils from CLL patients produced markedly higher levels of ETs compared to that in healthy subjects, and plasma from CLL patients increased the capacity of neutrophils from healthy subjects to release ETs (11). However, relatively little is known about the formation of ETs in APL. Our studies have shown that APL cells treated with ATRA or ATO also undergo this death process, producing ETs (12, 13). However, the role of ETs in APL-related bleeding is still unclear. PLTs are continuously activated in patients with malignant tumors, and abnormal PLTs activation has been detected in CML and CLL, suggesting that PLTs abnormalities may be a common phenomenon in leukemia (14, 15). PLTs are indispensable for the production of ETs *via* neutrophil adherence or secretion of soluble medium (16). However, the interaction between ETs and PLTs in APL is poorly understood.

We previously showed that pathological neutrophils, APL cells or NB4 cells, cause bleeding by damaging endothelium in APL (17). However, the role of ETs, a product of neutrophils, in endothelial injury- related hemorrhage is unclear in APL. Microvascular endothelial cells are the main barrier that separates blood stream from the underlying tissues. One of the hallmarks of endothelial integrity damage is increased permeability, which causes spontaneous bleeding in severe cases (18, 19). The structural integrity of the endothelium depends on adherence and tight junctions, which are mediated mostly by VE-cadherin (20, 21). ETs cause microvascular leakage passage by proteolysis of the intercellular junction protein and rearrangement contractile cytoskeleton (22, 23). Furthermore, ETs upregulate vascular permeability in model mouse of endotoxemia and transfusion-related acute lung injury (24, 25). Thus, it is attractive to speculate whether ETs play a role in APL blood cells leakage by destroying the endothelial integrity.

Here, we explored the differences in production of ETs by mature and immature neutrophils in APL, as well as the interaction between PLTs and ETs. Moreover, the effect of ATRA+ATO on the release of ETs was studied. Importantly, ETs cause endothelial damage, resulting in an increase in vascular permeability. Therefore, we also investigated the effects of ETs on bleeding by increasing endothelial permeability in APL. Our study provides a new treatment strategy for the intervention of APL-related bleeding during the induction period.

## Materials and Methods

### Patients

Peripheral blood was collected from 15 APL patients (newly diagnosed and ATRA+ATO induction treatment for 15 days) and 10 control volunteers. The subjects were admitted to the First and Second Affiliated Hospital of Harbin Medical University from December 2019 to October 2021. Each of the donors signed an informed consent form. APL was diagnosed based on clinical data, morphology, cytochemistry, immunology, cytogenetics and molecular pathology testing, or confirmation of the presence of the t (15, 17) (PML-RARα) fusion gene (26). **Table 1** summarizes main clinical characteristics of patients. This study was conducted in accordance with the Declaration of Helsinki and approved by the Ethics Committee of Harbin Medical University.

**Table 1:** Characteristics of patients with APL.

Characteristics	Pre-treatment (ND APL)	Post-treatment (IT APL)
Age, y	42.6 ± 13.34	
Gender, M/F	4/11	
WBC (*10^9^)	14.12 ± 16.75	5.39 ± 4.46*
Hb (g/L)	72.84 ± 13.35	65.05 ± 13.51
PLTs (*10^9^)	26.06 ± 14.19	32.46 ± 15.15
PT (s) (10-15 s)	15.34 ± 2.21	13.05 ± 1.15***
APTT (s) (20-40 s)	27.81 ± 3.49	30.13 ± 5.19*
Fibrinogen (g/L)	1.94 ± 1.37	2.95 ± 1.32*
D-dimer (mg/mL)	4.95 ± 4.22	2.05 ± 1.81
Hemorrhage (DIC+), n (%)	8 (53.33%)	

The main clinical and laboratory features of 15 APL patients with pre- and post- induction treatment (15 days). Data are presented as numbers (percentages) or median ± standard deviation (SD). APTT, activated partial thromboplastin time; Hb, hemoglobin; PLT, platelet; PT, prothrombin time; WBC, white blood cell. *P < 0.5, ***P < 0.005 vs Pre-treatment.

### Preparation of Neutrophils, PLTs, and Plasma

The purified methods of neutrophils and PLTs referred to the previous articles and were improved (27, 28). Neutrophils were obtained applying the neutrophil separation solution kit (TBD Sciences, Tianjin, China) following the manufacturer’s specifications. Briefly, venous blood of APL patients were collected using a vacuum tube containing 3.2% sodium citrate. Then, whole blood and neutrophil separation solution were added in a 15 ml tube at a volume ratio of 1:1 and centrifuged at 500 × g for 30 min. The neutrophil layer was slowly absorbed and added to the tube, and then 5 ml erythrocyte lysing solution was added and incubated for 5 min at room temperature. After incubation, centrifugation was repeated at 500 × g for 5 min until erythrocyte disappear. The supernatant was discarded, and 10 ml of physiological saline (PBS) was added before centrifuging at 500 × g for 5 min; the precipitates were neutrophils. Neutrophils were resuspended in RPMI 1640 and used immediately for analysis or *in vitro* stimulations.

PLTs were obtained by centrifugation. Briefly, fresh whole blood was centrifuged at 170 × g for 20 min to obtain platelet-rich plasma. Seventy percent of platelet-rich plasma was absorbed and centrifuged at 500 × g for 20 min. The obtained PLTs were resuspended in HEPES buffer for follow-up experiments.

Peripheral blood from the APL patients and healthy subjects were centrifuged for 10 min at 1000 g, and isolated supernatant was centrifuged for 20 min at 15000 g to obtain platelet-free plasma (PFP) (28). Plasma was obtained from fresh peripheral blood of patients and healthy subjects by centrifugation at 1500 × g for 10 minutes.

### Cell Culture

Human APL cell line NB4 cells from Meisen CTCC (Zhejiang, China) were maintained in RPMI 1640 medium containing 10% fetal bovine serum (FBS) and 1% penicillin-streptomycin. Human umbilical vein endothelial cells (HUVECs) from ScienCell (San Diego, CA, USA) were cultured in EC medium supplemented with 5% FBS, 1% growth supplement and antibiotics.

### Quantification of ETosis Markers

Plasma of ETosis markers, including myeloperoxidase-DNA (MPO-DNA) complexes and citrullinated histone 3 (CitH3), were quantified using enzyme-linked immunosorbent assays (ELISA) (Jingkang, shanghai). Briefly, diluted plasma was put in the 96-well plate coated with antibody and incubated at 37°C. After washing, TMB was added and incubated. After the addition of the stop solution, the absorbance at 450 nm was tested.

### Stimulation and Inhibition of ETosis

Neutrophils isolated from APL patients and control individuals were maintained in RPMI-1640 medium with or without 25 nM PMA, ATRA (1μM) (Aladdin, Shanghai, China), ATO (0.75 μM) (Sigma-Aldrich), ATRA+ATO or 5% plasma (from control individuals and APL patients) for 2 h. Neutrophils were cultured with PLTs from control or APL individuals in a ratio of 1:50 for 3 h to investigate the role of PLTs. Control PLTs were pretreated with 5% APL or control plasma for 30 min and incubated with control neutrophils for 3 h. For inhibition, PLTs were pretreated with anti-platelet factor-4 (PF4) antibody (Affinity) for 30 min.

### Immunofluorescence Test

Neutrophils or HUVECs were seeded in a 24-well plate and added with agonists (ETs, PMA, ATRA or ATO) or inhibitor (DNase 1, activated protein C (APC) and sivelestat). Then, the cells were fixed with 4% PFA for 15 min before being blocked. After then, samples were stained using corresponding primary antibody and fluorescent secondary antibody. For sytoxgreen staining, cells were stained with sytoxgreen for 10 min followed by 4% PFA fixation. The specimens were examined using a Zeiss LSM 800 (Carl Zeiss, Germany). The percentage of ETs cells that released cells was determined by evaluating 1000 cells using a double-blind experimental procedure.

### Flow Cytometric Analysis

Citrated blood freshly collected from APL and control individuals was stained with fluorescence labeled antibody for 30 min at 4°C. After fixing with 1% PFA, the samples were analyzed by a BD FACSCanto™ II flow cytometer (Becton, Dickinson and Company, USA). The detailed steps are in the **Supplementary Materials**.

### Endothelium Permeability Assay

HUVECs were added to the upper chambers of 3 μm inserts and grown to a confluent monolayer before being treated with ETs. An fluorescein isothiocyanate (FITC)-Dextran (70 kDa, sigma) was added to the upper chamber. The medium from the lower chamber was withdrawn and measured at 485/528 nm with a microplate reader.

### Endothelial Stimulation and Inhibition Assays

When HUVECs grew to 80% in 24-well plates, 0.5 μg/ml ETs were added to the medium without FBS for 4 h. ETs were pretreated with 100 U/mL DNase 1 (Thermo Fisher Scientific, USA), 100 nM Sivelestat (Sigma-Aldrich, S7198) or 100 nM APC (Med Chem Express) for 1 h in inhibition assays. The expression levels of vascular cell adhesion molecule-1 (VCAM-1), intercellular adhesion molecule-1 (ICAM-1), CD31, VE-cadherin and ZO-1 were analyzed using immunofluorescence test or western blotting.

### Western Blotting

HUVECs were harvested and lysed with RIPA buffer containing the proteinase inhibitor PMSF and phosphatase inhibitor cocktail. Proteins were separated using electrophoresis gel and transmitted to polyvinylidene difluoride membranes by electrophoresis. The membranes were then blocked with 5% non-fat milk, washed and incubated with primary antibodies before being treated with horseradish-peroxidase-conjugated secondary antibodies (ZSGB-BIO, Beijing, China). Visualization was performed with a Tanon 5100 Imaging System (Tanon, Shanghai, China).

### Animal Experimentation

The animal experiment was implemented according to the protocol approved by the Animal Care and Use Committee. Twenty aged 6-8 weeks SCID mice were fed with standard powdered rodent diet and autoclaved water at 22°C in a 12-hour light/dark cycle. The SCID mice were randomly distribute to four groups: control, APL, APL+ATRA+ATO and APL+ATRA+ATO+DNase 1. To establish *in vivo* experiments, SCID mice were injected with 1×10^6^ NB4 cells. The corresponding treatment was performed for three consecutive weeks. Before the mice were sacrificed, UVR irradiated the shaved back skin for 24 h. The tails of anesthetized mice animals were cut at 3-mm of the distal tip, which recorded bleeding time. The weight, appearance and behavior of the animals were evaluated every day. Mice were euthanized when they began to show the following symptoms: rapid weight loss, wrinkled hair, hunched posture, decreased activity, weak, diarrhea, head tilt and tremor. The liver, lung and other organs were fixed with 4% PFA, embedded in paraffin, sliced and subjected to pathological tests.

The methods for “scanning electron microscopy of ETs and ECs”, “assay for PLTs stimulation and inhibition”, and “red blood cells leakage and deposition assay” are presented in detail in the **Supplemental Methods**.

### Statistical Analysis

All results were expressed as mean ± standard deviation (SD). SPSS or GraphPad Prism 8.0 were used to analyze all data. Multiple groups were compared and analyzed by one-way ANOVA. Two pairs variables were analyzed by paired t-test. Statistical significance was determined when *P* < 0.05.

## Results

### Immature Neutrophils of APL Negatively Affect the Formation of ETs

**Figure 1:**
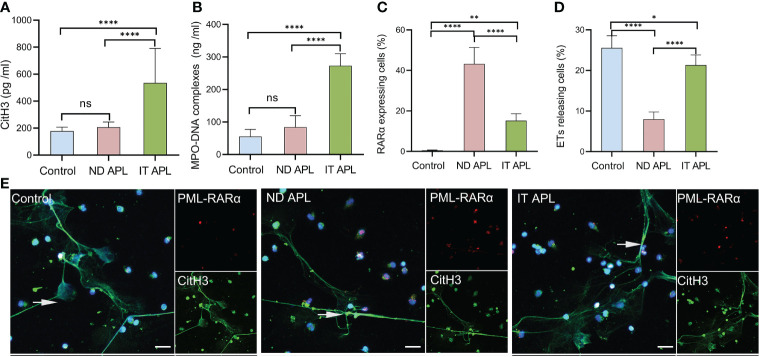
ETs release ability of mature and immature neutrophils in APL. **(A, B)** CitH3 and MPO-DNA levels in ND APL (n =15) and IT APL (n =15) patients’ plasma relative to controls (n =10) were detected by ELISA. **(C, D)** Percentage of PML-RARα-expressing cells and ETs-releasing cells. **(E)** Representative images showed ETs released by neutrophils from controls, ND and IT APL treated with 25 nM PMA. The undifferentiated APL neutrophils were identified by PML-RARα (red), ETosis was visualized by the co-localization of DNA (blue) with CitH3 (green). 20× objective. Scale bars indicate 20 μm. Arrows indicated ETs. All values are mean ± SD. ****P < 0.0001, **P < 0.01, *P < 0.05 and ns, not significant by one-way ANOVA or t-test.

**Figure 2:**
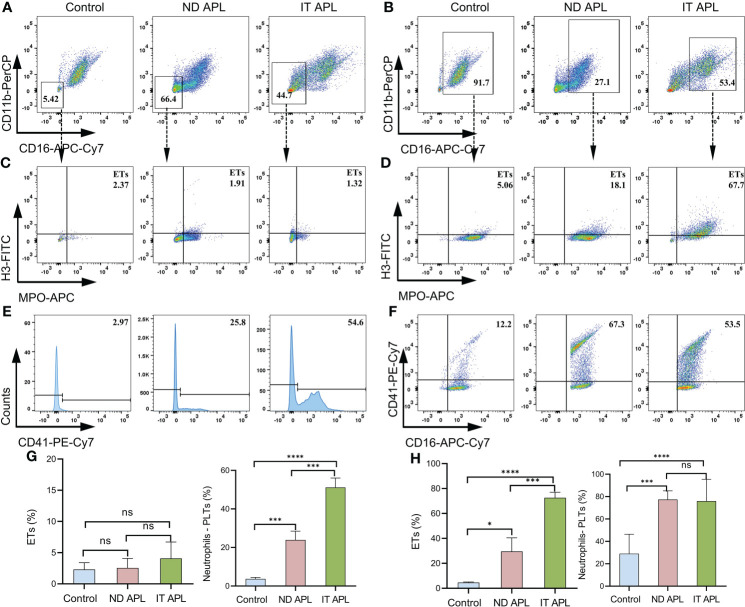
Flow cytometry images of ETs generation from neutrophils in APL. Whole blood from controls or patients was examined by flow cytometry. **(A)** CD16^-^ CD11b^-^ events identified as undifferentiated neutrophils. **(B)** CD16^+^ CD11b^+^ population represented differentiated neutrophils. **(C, D)** Representative dotplot of ETs in immature neutrophils (CD16^-^ CD11b^-^) or mature neutrophils (CD16^+^ CD11b^+^) from controls and APL patients. ETs were defined as CitH3^+^ MPO^+^ events. **(E, F)** Neutrophils–PLTs aggregates in undifferentiated neutrophils (CD41^+^events in CD16^-^ CD11b^-^ gates) and differentiated neutrophils (CD41^+^ CD16^+^ events in the CD16^+^ CD11b^+^ population) of APL. The numbers in the quadrants show percentage of gated events. **(G, H)** The graph shows the quantification of the flow cytometry data. All values are mean ± SD. ****P < 0.0001, ***P < 0.001, *P < 0.05 and ns, not significant by one-way ANOVA.

ETosis markers in plasma of control, newly diagnosed APL (ND APL) and induction therapy APL patients (IT APL) were examined to analyze ETosis of APL (**Figures 1A, B**). The levels of MPO-DNA complexes and CitH3 were significantly higher in IT APL plasma than in ND APL and control. However, there was no statistically obvious difference between ND APL and control in these ETs-specific markers. The undifferentiated APL neutrophils were identified based on the expression of PLM-RARα. ETosis was visualized by co-localization of DNA with CitH3 and quantified by measuring the percentage of ETs cells that releasing cells. First, purified neutrophils from control and APL patients were stimulated by 25 nM PMA to further verify the ability of patients’ neutrophils to release ETs. It was suggested that neutrophils from IT APL patients and control individual tended to release more ETs than neutrophils from ND APL (**Figures 1D, E**). Moreover, the number of ETs was lower in IT patients than control individuals. Compared with neutrophils from IT APL, neutrophils from ND APL expressed more PLM-RARα protein but released less ETs (**Figures 1C–E**). There was little PLM-RARα protein expression in control neutrophils. However, ETs releasing cells in ND patients displayed almost no red fluorescence, and PML-RARα expressing cells did not produce ETs (**Figure 1E**). It is preliminarily inferred that ETosis in APL patients is correlated to the degree of neutrophils differentiation. Then, fresh whole blood from healthy volunteers, ND APL and IT APL patients was analyzed using flow cytometry for ETosis of differentiated and undifferentiated neutrophils. The CD16^-^ CD11b^-^ population was defined as undifferentiated neutrophils (**Figure 2A**), while CD16^+^ CD11b^+^ population represented differentiated neutrophils (**Figure 2B**). ETosis differentiated neutrophils (MPO^+^ CitH3^+^ events in CD16^+^ CD11b^+^ population) were abundant in ND APL and IT APL patients (**Figure 2D**), while signs of ETosis were undetectable in undifferentiated granulocytes (CD16^-^ CD11b^-^) and healthy individuals (**Figure 2C**). The flow cytometry results verified the above conclusion that the ETosis ability of undifferentiated neutrophils was impaired. The proportion of ETs of differentiated neutrophils in IT APL samples was significantly higher than in ND APL patients, therefore induction therapy may enhance ETosis of neutrophils. Interestingly, neutrophils-PLTs aggregates increased in ND APL and IT APL compared to the control, including both undifferentiated neutrophils-PLTs (CD41^+^ counts in CD16^-^ CD11b^-^ population) and differentiated neutrophils-PLTs aggregates (CD16^+^ CD41^+^ events) (**Figures 2E, F**). The **Figures G, H** showed the quantification of the flow cytometry data.

### Effects of ATRA+ATO and Plasma From APL Patients on ETosis From Neutrophils in the Patients

**Figure 3:**
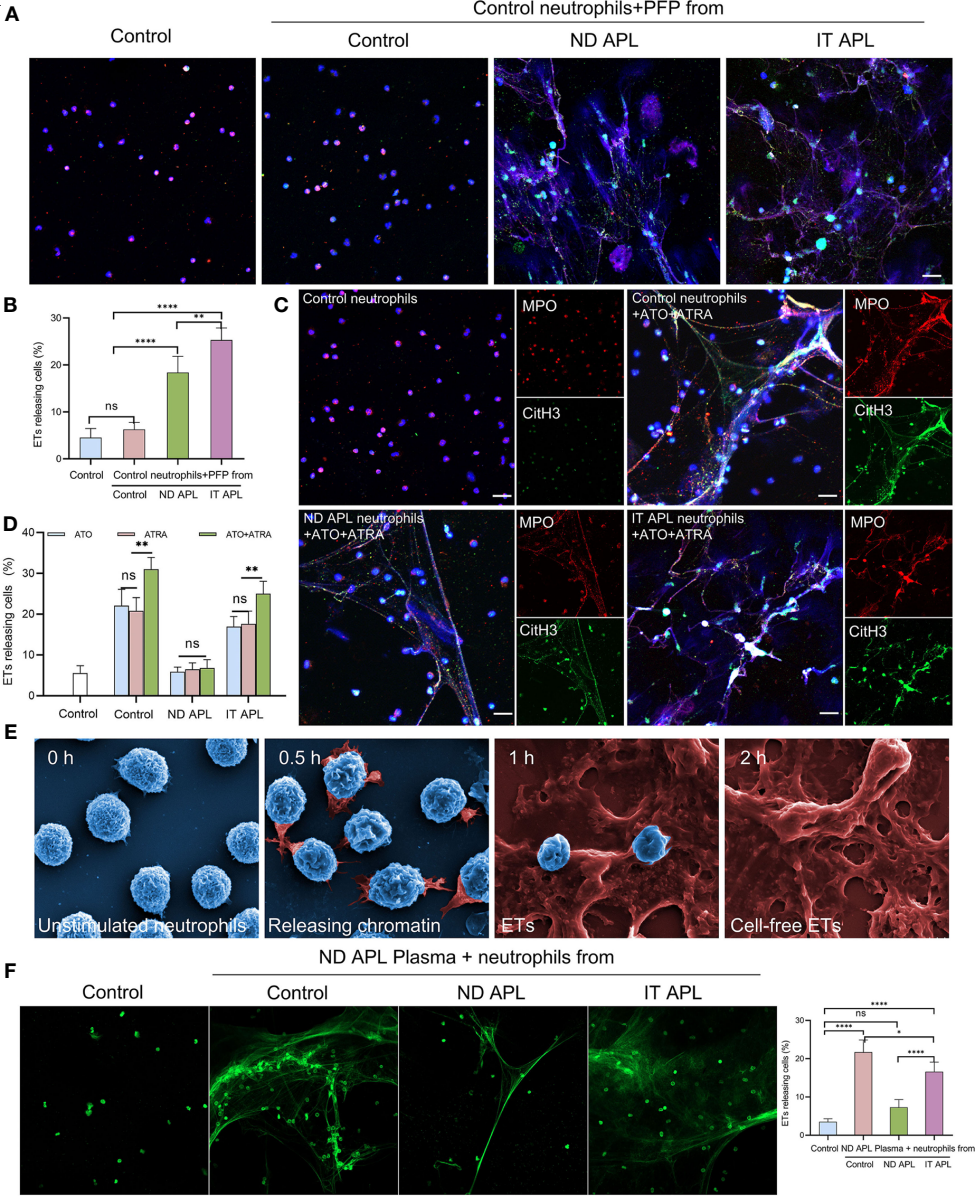
APL patient’s plasma and ATRA+ATO induces ETosis. **(A)** Representative images of ETs generation in control neutrophils treated with PFP from control, ND APL or IT APL samples. Red: MPO, Green: CitH3, Blue: DAPI. **(B)** Percentage of ETs-releasing cells. **(C)** ETs production of neutrophils extracted from control, ND APL or IT APL samples incubated with ATRA+ATO. **(D)** Percentage of ETs-releasing cells. **(E)** Electron microscopic images of neutrophils from IT APL exposed to ATRA+ATO. **(F)** Sytoxgreen images of ETs release in neutrophils from controls, ND APL and IT APL stimulated by ND APL plasma. 20× objective. Scale bar, 20 μm. All values are mean ± SD. ****P < 0.0001, **P < 0.01, *P < 0.05 and ns, not significant by one-way ANOVA.

To explore the factors that stimulate neutrophils to produce ETs in APL, neutrophils were extracted from the control individuals and stimulated with PFP from the control, ND APL and IT APL patients. The results showed that PFP of APL patients stimulated neutrophils to release ETs, and that PFP of IT APL was more effective than that of ND APL (**Figures 3A, B**). Since the frequent use of ATRA+ATO combined induction therapy in clinics, we treated neutrophils that were purified from control samples and APL patients with ATRA/ATO or ATRA+ATO. Whether ATRA, ATO or ATRA+ATO induced the formation of ETs in healthy individuals and IT APL patients, the combined treatment groups produced the most ETs (**Figures 3C, D**, the separate groups of ATRA/ATO are shown in **Figure S1**). It is worth noting that ETs were released at a lower rate in APL patients than in control individuals, and that the ND patients had the least number of ETs. The SEM analysis revealed that IT APL neutrophils that were exposed to ATRA+ATO lost cellular shape, expanded and released chromatin into extracellular space (**Figure 3E**). The body of APL patients is a complex environment. To verify whether there were any other factors affecting the release of ETs than induction drugs, we used plasma from ND APL patients to stimulate neutrophils from the control, ND APL and IT APL groups. The results revealed that the plasma from ND APL patients activated differentiated neutrophils to release ETs (**Figure 3F**).

### Activated Platelets Induce the Formation of ETs in APL

**Figure 4:**
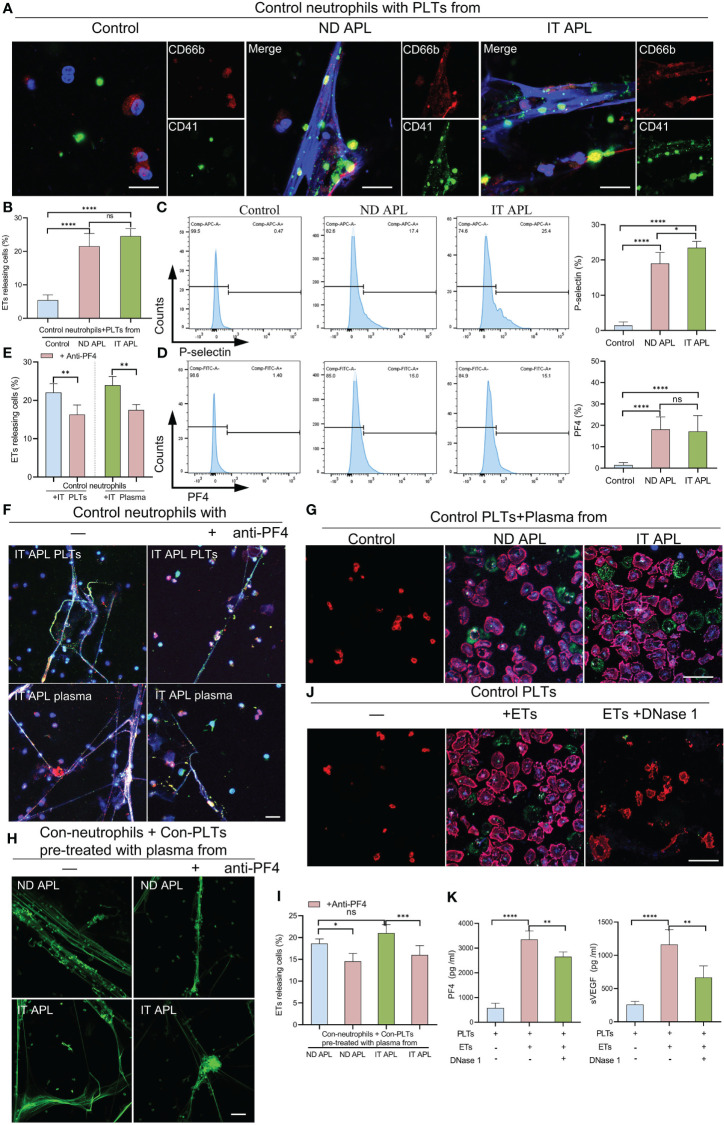
The interaction of PLTs and ETs in APL. **(A)** ETs generation by control neutrophils treated with PLTs isolated from control, ND APL and IT APL samples. **(B)** Percentage of ETs-releasing cells. **(C, D)** Flow cytometry analysis of P-selectin and PF4 on control, ND APL and IT APL PLTs. **(E)** Percentage of ETs-releasing cells. **(F)** Representative images of ETosis in neutrophils from controls supplemented with IT APL PLTs or plasma in the absence or presence of anti-PF4. **(G)** PLTs activation by control PLTs incubated with plasma form control, ND APL and IT APL. **(H)** ETs formation by control neutrophils supplemented with control PLTs pre-treated with plasma from ND APL and IT APL in the absence or presence of anti-PF4. **(I)** Percentage of ETs-releasing cells. **(J)** Representative images of platelets activation by ETs in the absence or presence of DNase 1. **(K)** ELISA detection of the concentration of PF4 and VEGF levels in the supernatant. F and H, 20× objective. A, G and J, 40× objective. Scale bar, 20 μm. All values are mean ± SD. ****P < 0.0001, ***P < 0.001, **P < 0.01, *P < 0.05 and ns, not significant by one-way ANOVA.

According to the flow cytometry results, neutrophils-PLTs aggregates increased in ND and IT APL granulocyte populations as compared to the control group. We sought to determine whether PLTs from APL patients have a role in ETosis. When the control neutrophils were treated with ND or IT APL PLTs, they significantly increased ETs release, whereas treating neutrophils with PLTs derived from control samples did not promote ETs formation (**Figures 4A, B**). Flow cytometry analysis revealed that PLTs from ND or IT APL had higher levels of P-selectin and PF4 than PLTs from the control group (**Figures 4C, D**). Moreover, PF4 increased was associated with activated PLTs. However, the toll-like receptor 4 (TLR4) and high mobility group box-1 (HMGB1) were not upregulated (**Figure S2**). Next, we performed inhibition experiments to verify the role of PF4 in PLTs-mediated ETs production in APL. When control neutrophils were incubated with IT APL PLTs and plasma, PF4 antagonists significantly reduced the release of ETs, indicating that PF4 plays a key role in PLTs inducing ETosis (**Figures 4E, F**). Furthermore, we discovered that ND- and IT APL-derived plasma was able to activate control PLTs *in vitro* compared with plasma from the control group (**Figure 4G**). Similar to ND or IT APL-derived PLTs, PLTs activated *in vitro* induced the production of ETs from control neutrophils (**Figures 4H, I**). The ETosis of PLTs activated by APL plasma decreased significantly when PF4 antagonists were administered. Next, we studied the effect of ETs on PLTs. ETs were extracted from IT APL patients and used in subsequent experiments. ETs activated PLTs from the control group and elevated P-selectin and phosphatidylserine (PS) expression on the surface of PLTs (**Figure 4J**), while DNase 1 inhibited PLT activation. Additionally, PF4 and vascular endothelial growth factor (VEGF) levels in the supernatant were increased (**Figure 4K**). While activated PLTs from APL induced neutrophils to release ETs, ETs also upregulated PLTs activation and released a variety of PLT factors.

### ETs Damage Endothelial Barrier Integrity

**Figure 5:**
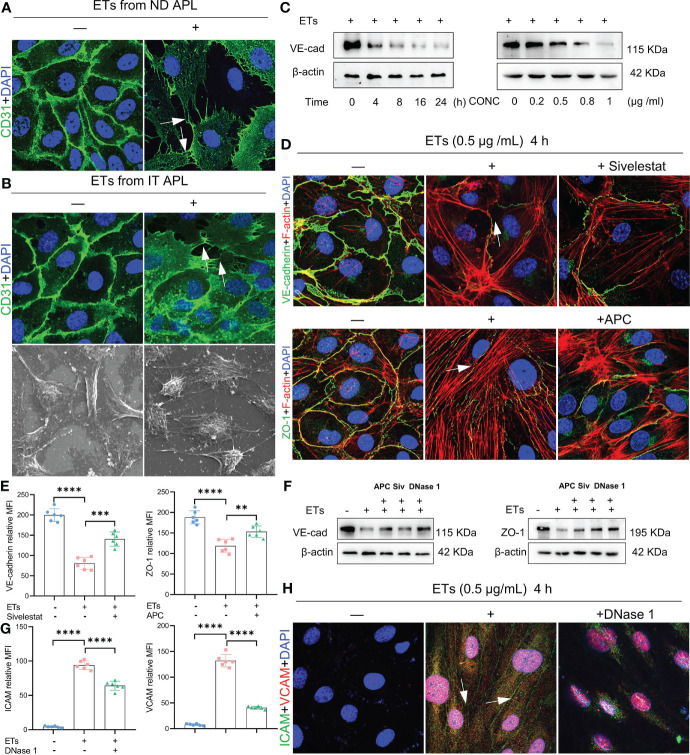
Effect of ETs on HUVECs barrier integrity. **(A, B)** The expression of CD31 and structure of HUVECs after incubating with ETs from ND APL or IT APL. The image showed retraction of cell margins and extension filopodia (arrows). **(C)** The expression of VE-cadherin of HUVECs treating with 0.5 μg/mL ETs for different time or various concentrations of ETs for 4 h.** (D)** Representative images of VE-cadherin and ZO-1 treated with 0.5 μg/mL ETs for 4 h. The intercellular junction protein decreased and retracted to produce gaps, and F-actin reorganized to form stress fibers (arrows). **(E)** The mean fluorescence intensity (MFI) above immunofluorescence results analyzed by Image J. **(F)** The expression of VE-cadherin and ZO-1 treated with 0.5 μg/mL ETs for 4 h. **(G, H)** The images and MFI of ICAM and VCAM. All values are mean ± SD. ****P < 0.0001, ***P < 0.001, and **P < 0.01 by one-way ANOVA.

ETs can activate endothelial cells and induce their death. Therefore, we evaluated the effects of ETs on ECs. First, we determined whether ETs from ND and IT APL exerted the same effects on HUVECs. ETs from both sources stimulated HUVECs to extend their filopodia (**Figures 5A, B**). Moreover, SEM results revealed that the morphology of ECs stimulated by ETs from IT APL (**Figure 5B**). Then, we selected ETs from IT APL patients for follow-up experiments. Expression levels of VE-cadherin were evaluated in HUVECs treated with different concentrations of ETs (at 4 h) or at different times (with 0.5 μg/ml ETs). It was found that ETs decreased the expression degrees of VE-cadherin in a concentration- or time-dependent manner (**Figure 5C**). Since ECs adhesion and integrity depend on intercellular junctional proteins, we further evaluated the expression levels of ZO-1 and VE-cadherin *via* immunofluorescence and western blotting. Compared to the controls, HUVECs treated with ETs exhibited thinner ZO-1 and VE-cadherin, tight junction proteins at cell-cell borders, reorganized F-actin to form a stress fiber and retracted EC borders resulting in gaps. Pretreatment of ETs with APC, Sivelestat or DNase 1 reduced ETs-mediated cytotoxicity (**Figures 5D–F**). Relative to untreated controls, HUVECs treated with ETs exhibited elevated expression levels of VCAM-1 and ICAM-1, however, these effects were counteracted by DNase 1 (**Figures 5G–H**). In conclusion, ETs change endothelial junctional proteins and actin-myosin distribution, thereby destroying endothelial integrity.

### ETs Increase the Leakage of HUVECs to RBCs

**Figure 6:**
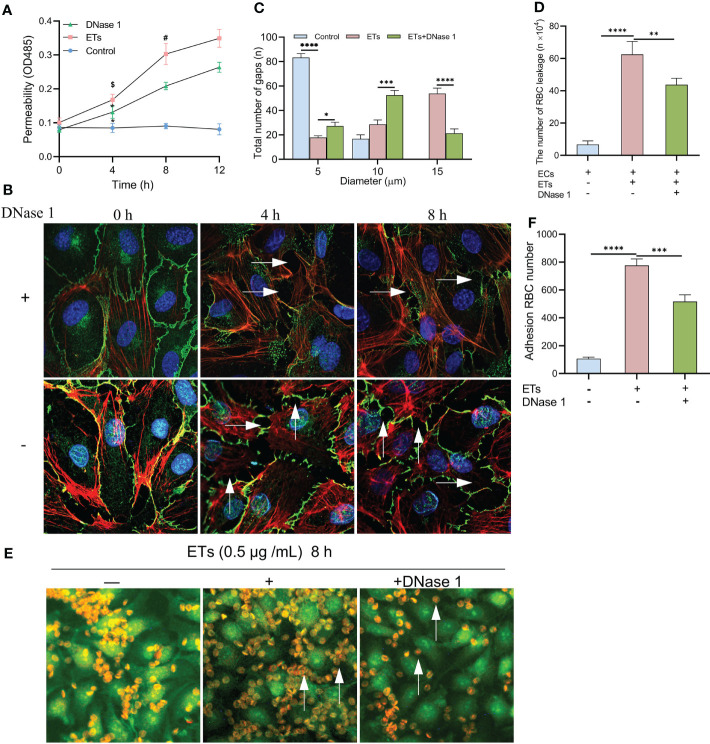
Increased HUVECs gaps allows RBCs to leakage. **(A)** The albumin permeability of ETs-treated monolayer ECs. *P < 0.05 vs 0 h, ^$^ *P* < 0.01 vs Control and ^#^ *P* < 0.05 vs DNase 1. **(B)** The loss of VE-cadherin expression and the augment of gap (arrows) in HUVECs treated with ETs increased with time, and DNase 1 mitigated this effect. **(C)** The number of intercellular gaps after ETs treatment for 8 h, from 100 gaps. **(D)** The number of RBCs leakage after incubation with monolayer HUVECs stimulated by ETs. The total number of added RBCs is 1×10^6^. **(E, F)** The experiment of RBCs deposition. RBCs was incubated with HUVECs treated with ETs for 8 h, and the number of RBCs deposition was analyzed. Arrows point to the RBCs deposited in the gaps. All values are mean ± SD. ****P < 0.0001, ***P < 0.001, **P < 0.01 and *P < 0.05 by one-way ANOVA.

Albumin permeability of ECs monolayer treated with ETs was raised in a time-dependent manner, and protein flux was higher than that of controls. Pretreatment with DNase 1 significantly reduced ETs-induced hyperpermeability (**Figure 6A**). ETs-treated ECs decreased the expression of VE- cadherin and enhanced gap sizes in a time-dependent manner. However, VE- cadherin loss and gap size enlargement were less relative to ETs group in DNase 1 treated ECs group (**Figure 6B**). Then, gaps between ECs were observed at 8 h under high power microscopy. In the ETs groups, about 82.25 ± 1.5% of gaps sizes more than 10 μm (including 10 μm), allowing RBCs (6-8 μm) to leakage. With DNase 1 pretreatment, the gap of 15 μm significantly reduced (**Figure 6C**). For transendothelial RBCs passage assay, RBCs were incubated with a monolayer endothelium in the upper layer of the transwell chamber. Compared to the untreated endothelial monolayer, RBC leakage values of ETs-treated ECs increased. However, RBC leakage reduced in the presence of DNase 1 (**Figure 6D**). In the RBCs deposition assay, due to intact endothelial spaces, RBCs floated over on the endothelium. After ETs stimulation, a large number of RBCs started to deposit in the endothelial gaps. Pretreatment with DNase1 decreased the number of deposited RBCs (**Figures 6E, F**). Therefore, ET-induced endothelial gap enlargement can lead to RBCs extravasation, which can be inhibited by DNase1.

### Inhibition of ETs Reduces Bleeding in SCID Mice Transplanted With NB4 Cells

**Figure 7:**
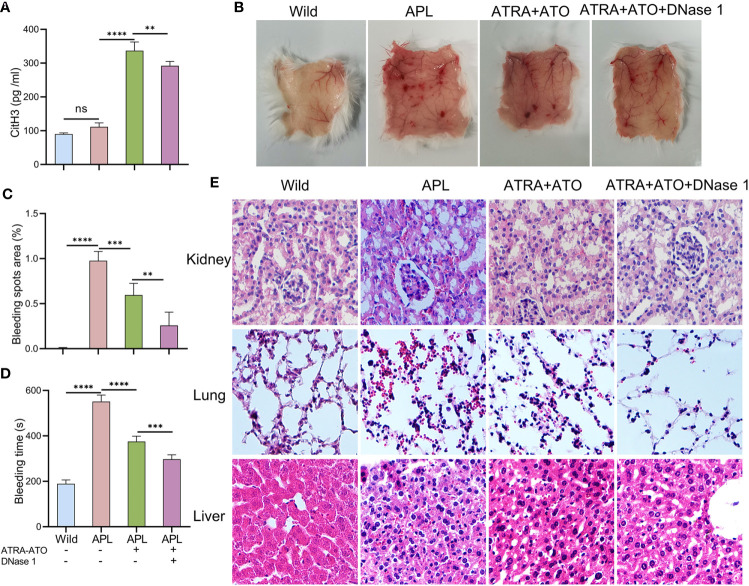
DNase 1 reduced bleeding in SCID mice transplanted with NB4 cells. **(A)** Plasma levels of CitH3 was measured in mice. **(B, C)** Representative images and bleeding spots area of back skins in mice for 24 h of UVR light exposure. **(D)** The bleeding time was tested in mice. **(E)** Representative HE images of kidney, lung and liver bleeding in different group. 20× objective. All values are mean ± SD. ****P < 0.0001, ***P < 0.001, **P < 0.01 and ns, not significant by one-way ANOVA.

NB4 cells were transplanted into SCID mice that were then monitored until they developed leukemia symptoms (**Figure S3**). After modeling, CitH3 levels were elevated in the peripheral blood of APL-ATRA+ATO mice, however, DNase 1 treatment decreased the concentrations of this index (**Figure 7A**). When mice were exposed to ultraviolet irradiation for 24 h, severe skin bleeding spots was observed in SCID mice transplanted with NB4 cells, while there were no bleeding spots in control mice. Treatment with a combination of DNase 1 and ATRA+ATO effectively markedly alleviated bleeding (**Figures 7B, C**) and shortened the bleeding time (**Figure 7D**). HE stains revealed bleeding in the liver, kidneys and lungs. Tissue sections showed hepatocyte swelling and turbidity, diffuse interstitial pneumonia and glomerular structure destruction. The above indices were improved in mice treated with ATRA+ATO or ATRA+ATO+DNase 1, and especially in the ATRA+ATO+DNase 1 group (**Figure 7E**). These results suggest that DNase 1 inhibited ETs-induced bleeding by protecting endothelial cells.

## Discussion

**Figure 8:**
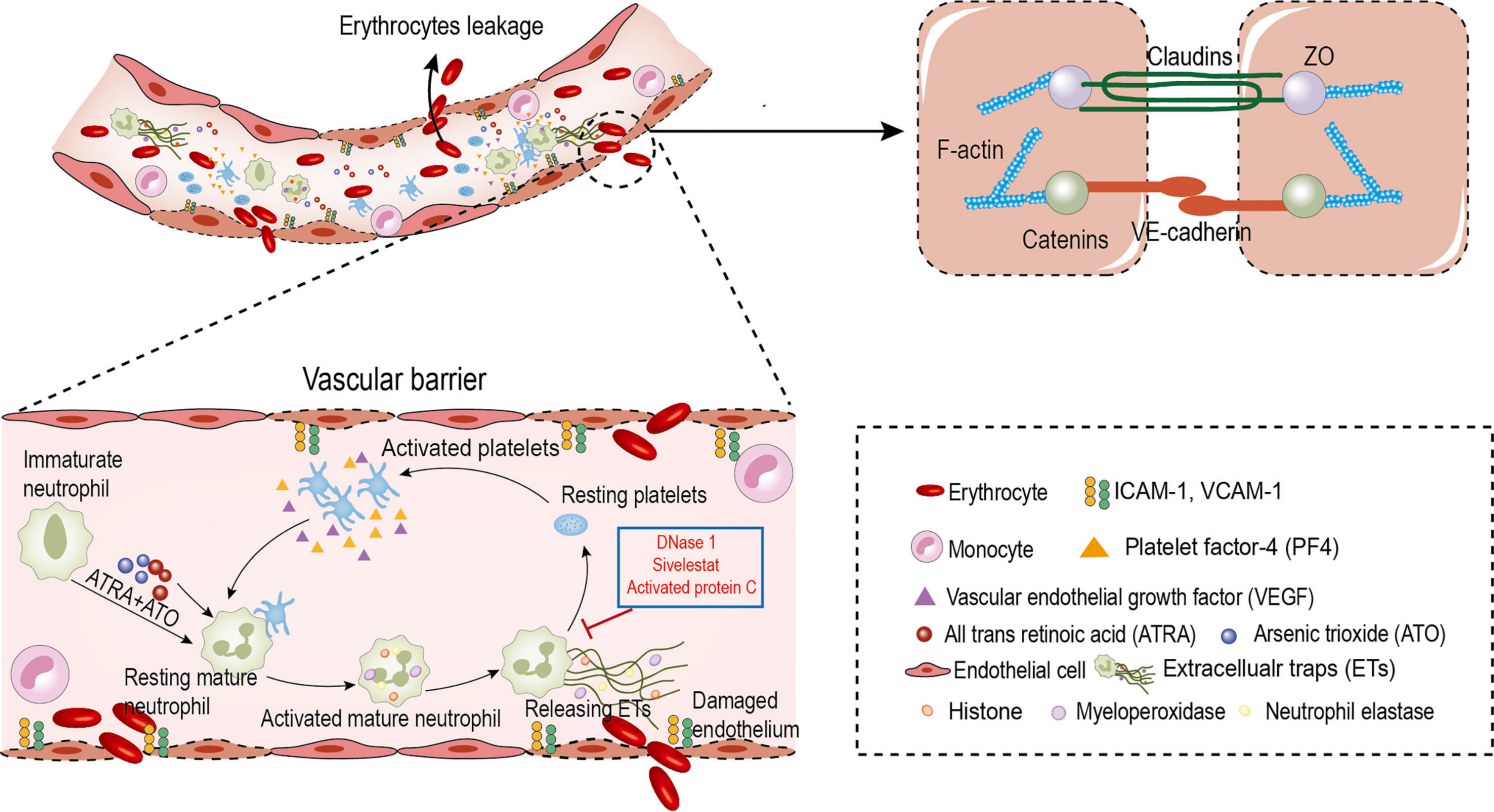
Circulating ETs contribute to erythrocyte leakage through damaged endothelial cells in APL. ATRA+ATO and platelets derived PF4 induce the differentiated neutrophils in APL to release ETs. ETs injury endothelial cells to increase intercellular gaps by destroying AJs and rearranging the contractile cytoskeleton. The increased gaps are large enough to allow red blood cells leakage. In addition, ETs activate platelets to form a positive feedback loop to aggravate this effect. DNase 1, APC and Sivelestat reduce erythrocyte leakage caused by endothelial injury.

We found that: first, the ability of immature neutrophils to release ETs was impaired, while mature neutrophils trended to produce ETs in APL; second, ATRA+ATO induction enhanced ETosis of mature neutrophils; third, PF4 derived from PLTs stimulated ETs formation, and ETs activated PLTs to secrete active substances, forming a positive feedback loop; finally, ETs trigger endothelial retraction and gap formation, leading to RBCs leakage. Moreover, the ETs inhibitor protected ECs from damage and alleviated APL bleeding (**Figure 8**).

ETs are involved in antimicrobial defense and in non-infectious diseases, including thrombosis, vasculitis and cancer. Elevated ETs levels have been detected in plasma and in tissues of many solid tumors (29–31). However, the significance of ETs in hematological tumors, especially APL, has relatively little been established. Neutrophils from CLL have been shown to be more prone to secreting ETs, and plasma from patients also stimulates neutrophils to release ETs (11). Similarly, ETs secretion from neutrophils was found to be increased upon PLTs-activating factor induction in CML mice (32). We found that unlike in chronic leukemias, plasma ETs markers in ND APL patients were only slightly highly elevated relative to those in healthy subjects, while plasma MPO-DNA and CitH3 levels were elevated in IT APL patients. Immunofluorescence and flow cytometry showed that ETosis abilities of immature neutrophils were impaired in APL, while ETs secreted by mature neutrophils increased. Magdalena Ostafin reported that immature neutrophils isolated from AML are characterized by a low ability to secrete ETs, which is in accordance with our findings (33). It is postulated that the insignificant trend of elevated ETs in ND APL patients is due to the high content of promyelocytes. Lukasova et al. found that neutrophil maturation determines the ability to release ETs, a property that is absent in incompletely differentiated neutrophils (34). Unlike APL, neutrophil counts and function are generally normal in untreated CLL patients (11, 35). Granulocytes of different maturation stages, which still have the ability of differentiation, have been reported to be in excess in CML (36). This may be the reason why neutrophil secretion of ETs in CLL and CML is not impaired. Moreover, we found that the proportion of ETs from differentiated neutrophils increased during APL induction treatment by ATRA+ATO. Whole blood flow cytometry analysis showed that differentiated neutrophils secreted more ETs in IT APL, compared to ND APL and controls. The results of PMA and ATRA+ATO stimulating neutrophils showed that ETs release in IT APL increased significantly compared to ND APL, but it was still less than that of normal control neutrophils. This is associated with neutrophil differentiation in patients during induction therapy, which partly restores the ability of differentiated neutrophils to release ETs.

Interactions of PLTs with neutrophils is indispensable in ETosis, a process that has been demonstrated in clinical and experimental contexts (37, 38). We found that proportions of neutrophil-PLTs aggregates in ND APL and IT APL patients were higher than in controls. Upon neutrophil-PLT aggregate formation, activated PLTs express or secrete various substances, such as TLR, HMGB1 and PF4, which are capable of stimulating ETs production (38–40). PLTs-derived PF4, one of the highest expressed proteins in PLTs, has been shown to induce ETs formation. A previous report has shown that PF4 participated in the formation of ETs in a mouse model of lung injury (41). We found that PLTs from ND and IT APL patients secreted more PF4, and antagonizing PF4 suppressed ETs secretion, consistent with the study by Agostina Carestia et al. (40). ETs can also initiate PLTs activation. Histone, a key ETs component, induces PLTs activation and aggregation (42). We confirmed that ETs activated PLTs derived from controls, increased the expression levels of activation markers (P-selectin and PS), and secreted PF4 as well as VEGF. DNase 1 alleviated ETs-induced PLTs activation. This positive feedback loop further magnifies PLTs activation and ETosis during the course of APL. VEGF affects liquid tumor exacerbation by directly acting on malignant cells or by indirectly effecting angiogenesis (43–45). VEGF is expressed on AML cells, and it plays an key role in hematological tumor growth (46). It regulates hematopoietic stem cell survival, changes clonal growth, inhibits apoptosis and promotes angiogenesis to enhance APL progression (47–49). These functions should be confirmed by further studies. In summary, for the first time, we elucidate on interactions between PLTs and ETs in APL. The central role of this positive feedback loop makes this process an attractive target for therapeutic intervention.

ETs play a role in the pathogenesis of vascular damage by inducing changes in endothelial barrier structures. Vascular endothelial integrity and permeability are regulated by interactions of intercellular junction molecules to allow minimal plasma fluid. Tight and adherence junctions (AJs) connections with actin cytoskeleton maintains ECs intercellular contact stability. We established that ETs-stimulated HUVECs had suppressed CD31 levels, thinned AJs proteins (ZO-1 and VE-cadherin) and formed actin stress fibers to enlargement intercellular gaps. Upon the stabilization of junctional proteins and actin cytoskeleton distribution by DNase 1, APC or Sivelestat, endothelial hyperpermeability and RBCs extravasation were markedly attenuated. Consistent with our results, it has been shown that CitH3 leads to barrier dysfunction and microvascular leakage by destroying AJs and rearranging the contractile cytoskeleton (22). Adhesion molecules are also involved in endothelial barrier regulation. In the absence of neutrophils, ICAM-1 increases endothelial permeability while blocking it reduces vascular endothelial injury and permeability (50). Neutrophils adhesion to ECs can lead to barrier damage, however, this is not necessary for hyperpermeability. Compared to adhesion damage of neutrophils to ECs, neutrophils derived ETs can cause endothelial barrier dysfunction without neutrophil adherence (51, 52). Importantly, ETs can resist shear flow and degradation in blood vessels for several hours (53–55). Therefore, these results lead us to believe that ETs plays a major role in ECs injury and blood cells leakage in APL. The results of our animal experiments showed that the bleeding symptoms of ATRA+ATO treated mice were alleviated compared with untreated SCID mice transplanted with NB4 cells. Although ATRA + ATO can ameliorate the bleeding of APL, SCID mice transplanted with NB4 cells still have bleeding symptoms due to the increase of ETs release after induction therapy. By interfering with ETs secretion, SCID mice transplanted with NB4 cells were prevented from bleeding. Thus, intervention of ETs release can protect vascular integrity to reduce APL bleeding. Our results form a basis for the development of new strategies for treating bleeding in APL.

A combination of ATRA+ATO is a feasible treatment option for APL patients, with long-term cure rate exceeding 90% (56). Compared to ATRA combined chemotherapy, ATRA+ATO regimen has a higher cure rate (57). In this study, combination of the two drugs induced undifferentiated neutrophils to mature and restore ETs secretion, which elevated ETs load in patients. Activated PLTs played an important role in ETs release during APL, and ETs activated PLTs in turn. This positive feedback loop further enhanced ETs production and exacerbated vascular endothelium burden in APL patients. Therefore, targeting ETs formation at the stage of APL induction therapy is a potential treatment strategy to reduce bleeding. Pretreatment of ETs with antibodies against varying components of ETs has been shown to reduce ETs-mediated cytotoxicity by varying degrees. APC is a serine protease, cleave histone to degrade toxicity in ETs, recombinant human APC has been approved by FDA for sepsis (58). Another abundant protein component of ETs is neutrophil elastase, and Sivelestat treatment, which can be used to inhibit neutrophil elastase in patients with acute lung injury (59). DNase 1, which degrades extracellular chromatin, has been clinically used to treat cystic fibrosis (60). We pretreated ETs with these three inhibitors, and they were shown to reduce endothelial toxicity of ETs. Clinical applications of DNase 1 and sieverestat makes it possible to inhibit ETs formation in APL patients.

In conclusion, the function of ETs released by APL immature neutrophils was impaired. Induction therapy triggered neutrophil differentiation and restored ETs release, which may also be associated with activated PLTs. Moreover, ETs can damage the endothelium, leading to RBCs leakage and bleeding, which can be alleviated by administration of ETs antagonists. Since bleeding is the main cause of death in the induction period, future treatment strategies should focus on DNase I combined with ATRA+ATO to expedite ETs degradation to further reduce the risk of haemorrhage.

## Data Availability Statement

The raw data supporting the conclusions of this article will be made available by the authors, without undue reservation.

## Ethics Statement

The studies involving human participants were reviewed and approved by the Ethics Committee of Harbin Medical University. The patients/participants provided their written informed consent to participate in this study. The animal study was reviewed and approved by Animal Care and Use Committee of Harbin Medical University.

## Author Contributions

YW and JS designed the research, performed experiments, analyzed data, made the figures and wrote the paper. JS analyzed data and revised the manuscript and SF provided funding support. CW performed experiments and analyzed data. NZ performed experiments and made the figures. HY performed experiments and excellent technical assistance. SF contributed on confocal microscopy and the flow cytometry experiments. All authors contributed to the article and approved the submitted version.

## Funding

This study was supported by grants from the National Natural Science Foundation of China (82170262 and 81870353).

## Conflict of Interest

The authors declare that the research was conducted in the absence of any commercial or financial relationships that could be construed as a potential conflict of interest.

## Publisher’s Note

All claims expressed in this article are solely those of the authors and do not necessarily represent those of their affiliated organizations, or those of the publisher, the editors and the reviewers. Any product that may be evaluated in this article, or claim that may be made by its manufacturer, is not guaranteed or endorsed by the publisher.
