# Supplementary material for: Extracellular Traps Increase Burden of Bleeding by Damaging Endothelial Cell in Acute Promyelocytic Leukaemia
Source: Front Immunol. 2022 Apr 11;13:841445. doi: 10.3389/fimmu.2022.841445 (PMC9035902; doi:10.3389/fimmu.2022.841445)
Supplement: Supplementary file 1 [file DataSheet_1.doc]

**Supplementary Materials and Methods**

**Reagents**

Mouse anti-myeloperoxidase (MPO) mAb (ab25989), rabbit anti-citrullinated histone 3 (CitH3) mAb (ab5103), anti-CD31 mAb (ab9498), anti-ZO-1 mAb ( ab216880), anti-VE mAb (ab33168), anti-rabbit Alexa Fluor (AF) 488 antibody (ab96899) were obtained from Abcam (Cambridge, Cambridge, UK). Mouse anti-intercellular adhesion molecule-1 (ICAM-1) mAb (60299-1-Ig), rabbit anti-vascular cell adhesion molecule-1 (VCAM-1) mAb (11444-1AP), anti-mouse AF 488 antibody and anti-rabbit AF 594 were from Proteintech (Wuhan, China).

**Generation and isolation of** **extracellular traps (ETs) structures**

Neutrophils were extracted using the neutrophil separation solution kit as previously described. Then, neutrophils were stimulated with 500 nM PMA for 4 h at 37°C. The medium was gently sucked and discarded, leaving the ETs and neutrophils at the bottom, adding cold PBS and centrifuging at 500 × g for 5 min. The supernatant was centrifuged again at 15000 × g for 15 min at 4°C. The precipitate was resuspended in 200 μl PBS, and the concentration of cell free-DNA was determined using the trace DNA meterthe (BioSpec-nano, Shimadzu, Japan), before being stored at -20°C.

**Flow cytometric analysis**

Fresh blood derived from acute promyelocytic leukemia (APL) patients and control individuals were stained for 30 min at 4℃ with Percp-Cy5.5-labeled anti-CD11b (Biolegend, San Diego, CA, 301328), APC-Cy7-conjugated CD16 (Bioledgend, 302018), PE-Cy7-conjugated CD41 (Bioledgend, 359812), anti-mouse MPO (Abcam, ab25989), and anti-rabbit CitH3 (Abcam, ab5103). After washing with PBS, samples were incubated with anti-rabbit AF 488 antibody (Abcam, ab15077) and anti-mouse AF 647 (Abcam, ab150115) for 30 min. Identification of ETosis and neutrophil-PLTs aggregates was performed as previously reported (1). Briefly, ETosis was characterized by the presence of both FITC and APC fluorescence, whereas neutrophil-PLTs aggregates were identified by an increase in both PE-Cy7 and APC-Cy7 fluorescence. To analyse the expression of P-selectin, platelet factor-4 (PF4), high mobility group box-1 (HMGB1) and the toll-like receptor 4 (TLR4) on platelets, fresh whole blood incubated with PerCp-Cy5.5-labeled anti-CD41 (Bioledgend, 303720), APC-conjugated anti-CD62P (Biolegend, 304910), anti-rabbit-PF4 (Affinity), PE-labeled anti-HMGB-1 (Bioledgend, 651404) and PE-labeled anti-TLR4 (Bioledgend, 312805) from controls and APL patients, respectively. The samples were incubated with anti-rabbit AF 488 antibody ( Abcam, ab15077). After fixing with 1% paraformaldehyde (PFA), the samples were analyzed by a BD FACSCanto^TM^ II flow cytometer (Becton, Dickinson and Company, USA).

**Scanning electron microscopy of ETs and ECs**

Human umbilical vein endothelial cells (HUVECs) cultured on coverslips were treated with ETs for 4 h in 24-well plates. Neutrophils, grown on coverslips, were stimulated with all-trans retinoic acid (ATRA) +arsenic trioxide (ATO) for 2 h. After washing thoroughly, the samples were fixed with 2.5% glutaraldehyde. After washing with Na-cacodylate HCl, specimens were treated with OsO_4_ and dehydrated with a gradient of ethanol concentrations. After drying at the critical point, the samples were coated with thick platinum layer. Finally, the photographs were captured by the S-3400 N electron microscope (Hitachi Co., Ltd.).

**Assay for PLTs stimulation and inhibition**

PLTs from the control group were plated on slides and cultured for 1 h with ETs from induction therapy APL patients (IT APL). Isolated ETs were pretreated with DNase1 before culturing with PLTs for the inhibition experiment. In the plasma stimulation test, PLTs from healthy individuals were incubated for 1 h with plasma from control, newly diagnosed APL patients (ND APL) and IT APL. After staining phosphatidylserine (PS) with FITC-lactadherin (Haematologic Technologies, Essex Junction, VT), PLTs were fixed with 4% PFA, blocked 5% bovine serum albumin and incubated with anti-rabbit-CD62P (1:500, NB100-65392SS, Novus biologicals, USA) and anti-mouse-CD41 (1:500, NBP2-50443, Novus biologicals) primary antibodies. Subsequently, all samples were incubated with anti-mouse Alexa Fluor (AF)- 594 (Proteintech, Hubei, China) and AMCA -conjugated Affinipure goat anti-rabbit IgG (H+L) ( Proteintech) secondary antibodies.

**Red blood cells leakage and deposition assay**

For red blood cells (RBCs) leakage test, HUVECs were seeded into insert well, grown to a confluent monolayer and stimulated with 0.5 ug/ml ETs for 8 h. The RBCs were isolated from healthy individuals, mixed with physiological saline and added to the HUVECs for 20 min. The leaked RBCs was determined utilizing a cell counting board. For RBCs deposition assay, HUVECs labeled with Celltracker Green CMFDA (YEASEN, Shanghai, China) were cultured in 24-well plates and stimulated with ETs at grew to a monolayer. The RBCs were stained with Celltracker CM-DiI (YEASEN) before being cultured with HUVECs for 20 min. The image of deposited RBCs was assayed and captured by a confocal microscope.

**References**

1. Perdomo J, Leung H H L, Ahmadi Z, Yan F, Chong J J H, Passam F H, et al. Neutrophil activation and NETosis are the major drivers of thrombosis in heparin-induced thrombocytopenia. Nat Commun (2019) 10(1): 1322. doi:10.1038/s41467-019-09160-7

**Supplementary Figure legend**

**Figure S1 ATRA or ATO induces neutrophils form APL to release ETs**Representative immunofluorescence images of ETs release in neutrophils from controls, ND APL and IT APL induced by ATRA or ATO. Red: MPO, Green: CitH3, Blue: DAPI.

**Figure S2 The level of HMGB1 and TLR4 from APL PLTs.**Representative flow cytometry plots of HMGB1 and TLR4 on control, ND APL and IT APL PLTs. The quantification of the flow cytometry data shown on the right. All values are mean ± SD. ns = not significant by one-way ANOVA.

**Figure S3 PML-RARα protein was expressed in spleen tissue of APL model mouse.**
