# Supplementary figures and images for: Extracellular Traps Increase Burden of Bleeding by Damaging Endothelial Cell in Acute Promyelocytic Leukaemia
Source: Front Immunol. 2022 Apr 11;13:841445. doi: 10.3389/fimmu.2022.841445 (PMC9035902; doi:10.3389/fimmu.2022.841445)

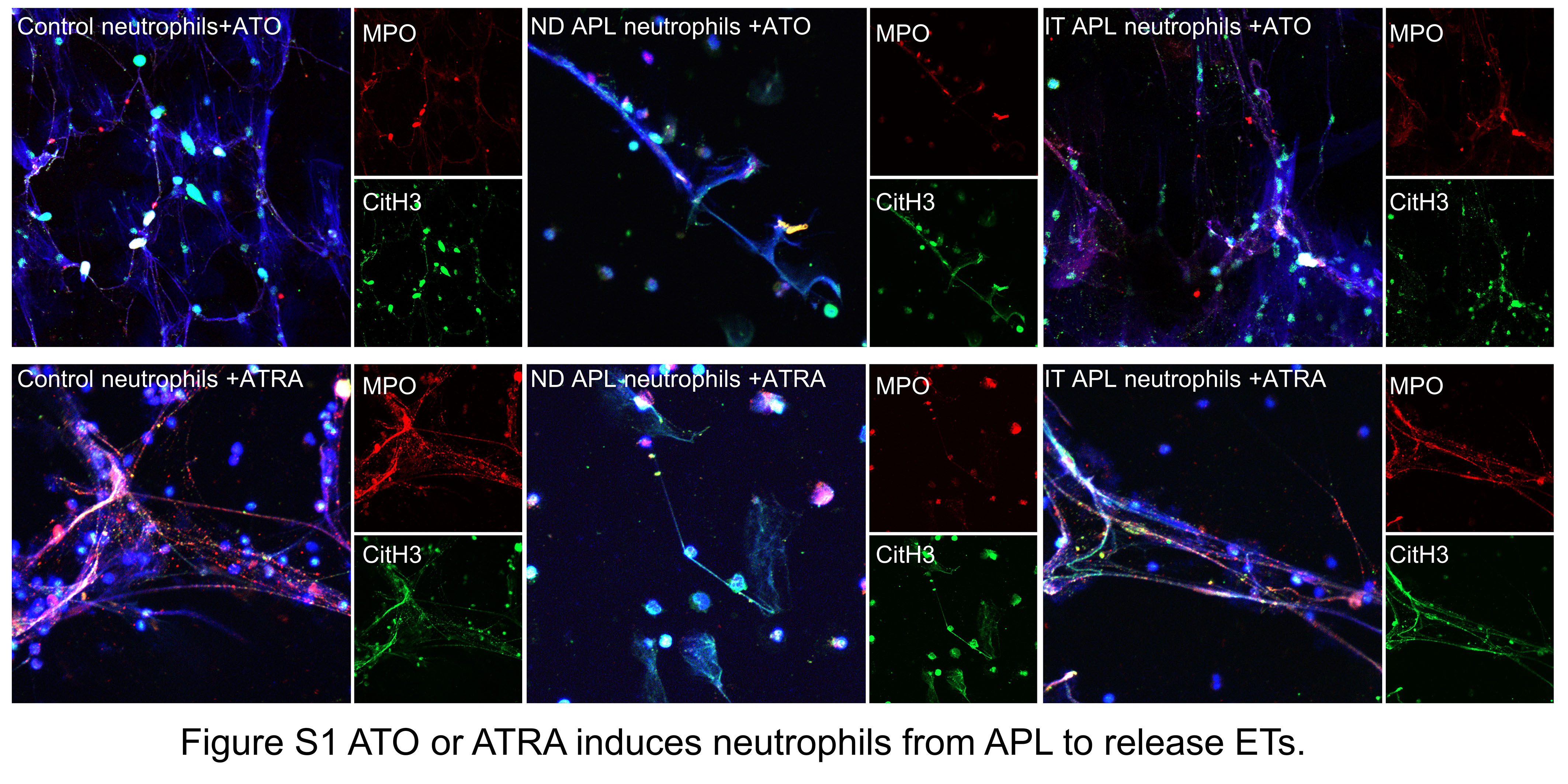

Supplement: Supplementary file 2 [file Image_1.jpeg]

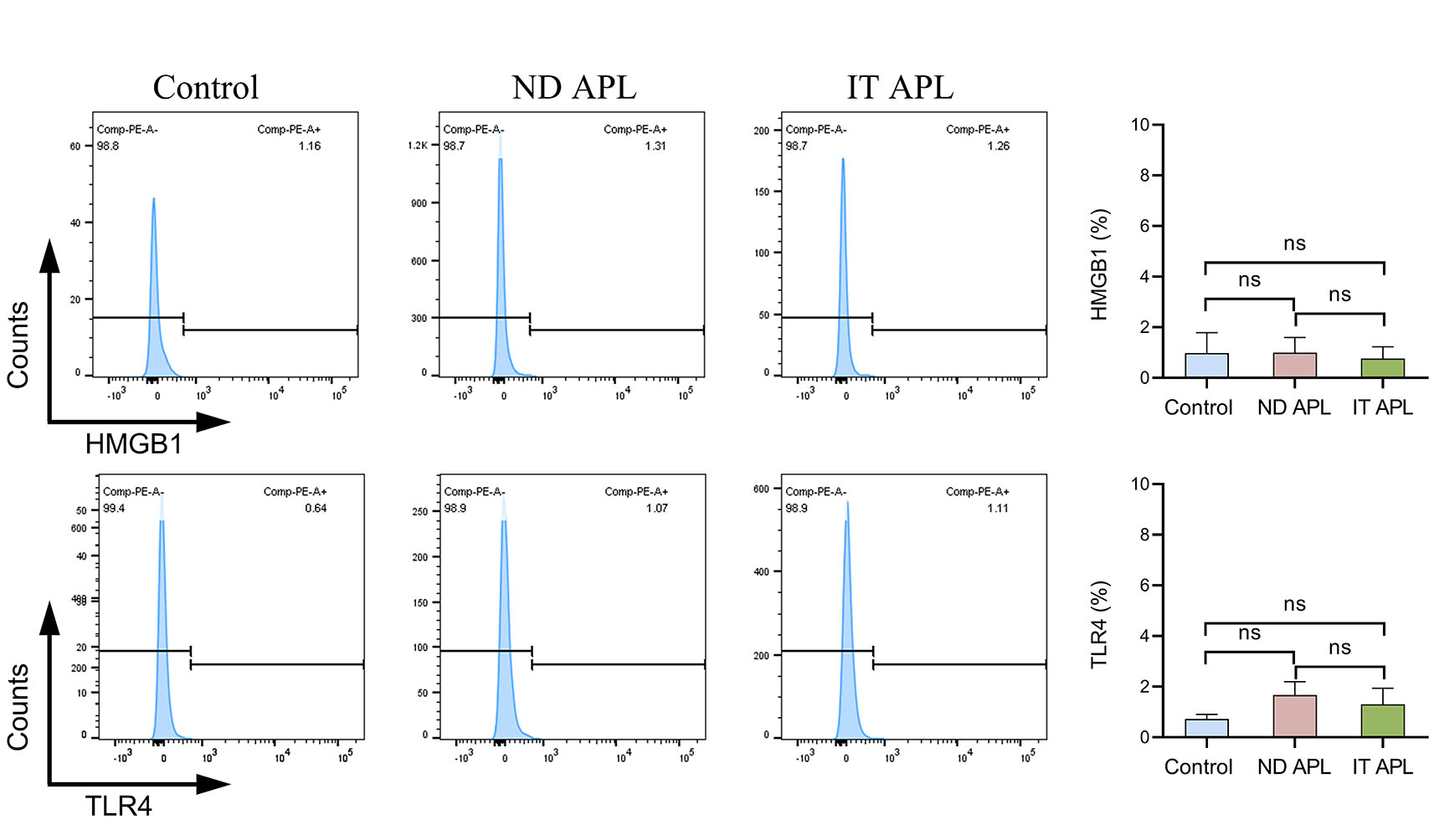

Supplement: Supplementary file 3 [file Image_2.jpeg]

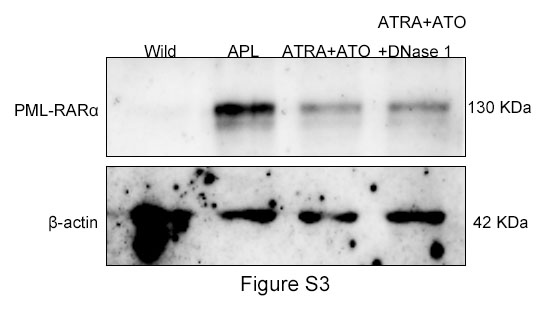

Supplement: Supplementary file 4 [file Image_3.jpeg]
